# The Occurrence, Sources and Spatial Characteristics of Soil Salt and Assessment of Soil Salinization Risk in Yanqi Basin, Northwest China

**DOI:** 10.1371/journal.pone.0106079

**Published:** 2014-09-11

**Authors:** Zhang Zhaoyong, Jilili Abuduwaili, Hamid Yimit

**Affiliations:** 1 State Key Laboratory of Desert and Oasis Ecology, Xinjiang Institute of Ecology and Geography, Chinese Academy of Sciences, Urumqi, China; 2 University of the Chinese Academy of Sciences, Beijing, China; 3 Key Laboratory of Xingjiang Arid Land Lake Environment and Resource, Xinjiang Normal University, Urumqi, China; NERC Centre for Ecology & Hydrology, United Kingdom

## Abstract

In order to evaluate the soil salinization risk of the oases in arid land of northwest China, we chose a typical oasis-the Yanqi basin as the research area. Then, we collected soil samples from the area and made comprehensive assessment for soil salinization risk in this area. The result showed that: (1) In all soil samples, high variation was found for the amount of Ca^2+^ and K^+^, while the other soil salt properties had moderate levels of variation. (2) The land use types and the soil parent material had a significant influence on the amount of salt ions within the soil. (3) Principle component (PC) analysis determined that all the salt ion values, potential of hydrogen (pHs) and ECs fell into four PCs. Among them, PC1 (C1^-^, Na^+^, SO_4_^2-^, EC, and pH) and PC2 (Ca^2+^, K^+^, Mg^2+^and total amount of salts) are considered to be mainly influenced by artificial sources, while PC3 and PC4 (CO_3_^-^ and HCO_3_^2-^) are mainly influenced by natural sources. (4) From a geo-statistical point of view, it was ascertained that the pH and soil salt ions, such as Ca^2+^, Mg^2+^ and HCO_3_^-^, had a strong spatial dependency. Meanwhile, Na^+^ and Cl^-^ had only a weak spatial dependency in the soil. (5) Soil salinization indicators suggested that the entire area had a low risk of soil salinization, where the risk was mainly due to anthropogenic activities and climate variation. This study can be considered an early warning of soil salinization and alkalization in the Yanqi basin. It can also provide a reference for environmental protection policies and rational utilization of land resources in the arid region of Xinjiang, northwest China, as well as for other oases of arid regions in the world.

## Introduction

Soil salinization is a global problem and it is a potential environmental problem in all continents with the exception of unassessed Antarctica. Soil saline levels are found within a wide range, and soil salinization occurs in much of the waterfront, arid and semi-arid zones of more than 100 countries and regions [1]–[3]. According to statistics done by the United Nations Educational, Scientific and Cultural Organization (UNESCO), and the Food and Agriculture Organization (FAO), salinized soil covers an area of about 9.543×10^6^ km^2^ on Earth [4]–[6]. In China alone, the area of salinized soil is about 3.693×10^5^ km^2^, which accounts for about a third of the total arable land [7]–[8]. The area of salinized soil in the oasis basin of Xinjiang in northwest China is about 1.05×10^4^ km^2^, which accounts for 33.4% of the total land in this area and research has found that the salinity of this area is trending upwards [9], [10]. Soil salinization restricts agricultural development, especially when sustainable agricultural development and environmental quality improvement strategies are being considered. Studies have found that when the salt ions in the soil attain 8 g.kg^−1^, they can greatly harm and even kill crops in farmland [11], [12].

In oases of arid regions of northwest china, the environment is so weak, including a lack of precipitation and high envapotion, that economic activities such as fishing, agriculture, forestry and grassland farming of the oases have been strongly limited, especially for agriculture [13]–[15]. Therefore, it is necessary to identify the distribution characteristics, sources of the soil properties, such as salt ions, potential of hydrogen (pH) and electrical conductivity (EC), and also the status and causes of salinization of the land of the oasis, in order to provide a scientific basis for protection of the soil that sustains land plants.

Multivariate analyses and other statistical methods have been widely applied in studies to determine the sources of elements found in soil, such as total soil salt content and heavy metals [16]–[18]. The spatial variation model and spatial distribution are used to make a hazard risk map of soil salt properties in regions of interest. Correlation analysis, principal component (PC) analysis and cluster analysis are classic methods used to identify the natural and man-made sources of salt ions and to simplify data. Additionally, use of the comprehensive index results in a class of data with high correlations that better reflect the associations between the data.

The Geostatistical Analyst is based on GIS technology [19], [20]. Among these, the ordinary kriging is the most widely used one in the study of soil salt distributions [21], [22], [29]. In recent years, the Geostatistical Analyst method has been used in the field of hydrology and water resources, including studies of groundwater pollution risk, water potential research and spatial distribution of soil salinization in arid land [23]–[25], [28], [32]. Since the 1990s, geostatistical methods have been widely used to study spatial variability characteristics of soil salt properties (salt ions, EC and pH). Sylla et al. [26] studied the spatial variation characteristics of soil salt content of an agricultural ecosystem under different scales in West Africa. Ammari et al. [27] studied the soil salinity changes in the Jordan Valley and the potential threat against the sustainable irrigated agriculture. In China, Bai et al. [30] researched the spatial variation characteristics and composition of soil salt content in Huang Huai Hai plain, northeast China and found that the average influential range of the soil salt content was higher than 200 km, indicating the salt content of the soil is mainly gathered in a large area of the regions. In Xinjiang in northwest China, Lin et al. [33] researched the spatial variation characteristics of soil salt in the Wei Gan He irrigated area and found the agricultural irrigation has resulted in serious soil salinization in this area and these deserve serious attention.

In arid regions, oases in basins are the main places where humans live and life can survive [31]. Therefore, it is important to understand the spatial distribution characteristics of the soil salt properties, including total salt content, salt ions, pH and EC. A quantitative grasp of soil salinity levels could serve as a reference and a basis for maintaining soil quality, which would help to effectively control the human pollution and develop the regional economy in a reasonable and orderly fashion [34], [35]. However, previous research has focused on rapidly developing areas, such as coastal plains and large irrigation areas in eastern china and elsewhere of the world with the purpose of assessing land usability, environmental effects and soil salinization risk [36]–[38]. Since the 1990s, implementation of the “western development policy of China” has led to prodigious economic development in many oases in Xinjiang, and the agriculture in these regions has undergone rapid progress. However, the rational irrigation of the agriculture, lack of precipitation and high envapotion of these regions have negatively influenced soil salt properties, resulting in increased soil salt contents, ECs and pHs, which can result in serious soil salinization [39]. Unfortunately, research on the soil salt property distribution characteristics and soil salinization risk assessment in the oases of arid regions of northwest of China is lacking.

The Yanqi basin is a typical oasis in a basin in the southern Tianshan Mountains, Xinjiang in northwest China. Since the 1990s, both the implementation of the “western development policy of China” and the development policy made by the Xinjiang Province, China, have led to prodigious economic development in the Yanqi basin, but regional economic development and associated human activity have left the current ecological environment fragile [39], [40]. Together with economic development, the blind expansion of farmland and unrestrained surface water irrigation led to a rise in groundwater and an increase in soil salinization in the basin oasis. 64.12% of the area experienced mild soil salinization, 8.25% had moderate salinization, and 27.07% had severe salinization. Research has shown that excessive use of water resources by agriculture has made the soil salinization status severe and decreased agricultural production [41].

After a basic analysis of land use and soil parent materials types in the area, we created land use and soil type geological maps, and, using ArcGIS 10.0 software and combining the grid sampling method with 3S technology, we made sampling points to get soil samples across the whole area. We evaluated the soil salt properties in different land use types in the laboratory, and assessed the soil salinization status and the cause in the Yanqi basin. Then ordinary kriging of Geostatistical Analyst method was used to reveal the spatial distribution characteristics of the soil salt properties in this region. Then by combining these properties with the climate, precipitation, evaporation and temperature, we assessed the soil salinization risk of this area. From this we can provide helpful proposals to prevent the environmental risks that could lead to soil salinization in this area. This research can serve as a helpful reference for environmental protection in this region and for soil salinization prevention in arid regions of northwest China.

## Materials and Methods

### Study area

The area studied in this work is a desert basin oasis in the arid region of northwest China including four counties in the Yanqi basin: Yanqi County, Hejing County, Bohu County and Heshuo County. This region lies within the geographical coordinates of 85°50′–87°50′E and 41°40′–42°20′N with a length of about 85 km from north to south and width of 130 km from east to west, totaling an area of about 723100 km^2^. The terrain slopes up from the northwest down to the southeast. The northwest is mountainous and the south is low-lying desert that is 1050–2000 m above sea level. The western area has extensive intrusive rock and metamorphic rock from the *Proterozoic era*, *Neoproterozoic* and *Cenozoic*. Weathering of this rock results in brown earth soil, acidic rocky soil and an acidic soil skeleton in the west. The east is primarily made up of quaternary sediments, which form *Takyic* (Calcisols), *Chemic* (Phaeozems), *Stagnic* (Gleysols), *Irragric* (Anthrosols), *Fragic* (Arenosols), *Eutric* (Gleysols) and *Yemic* (Solonchaks) [42]. The area researched is in a continental desert climate temperate zone with an annual mean temperature of 14.6°C, 186 frost-free days per year, and 50.7–79.9 mm of annual precipitation. By calculating the potential evaporation (*ET_0_*) by the method of Hargreaves (1985) [43], we then got the annual average potential evaporation of this area as 2438.9 mm, the ≥10°C active accumulated temperature 3414.4–3694.1°C and an annual average relative humidity of 72%.

### Soil sampling and analyses

In order to perform a basic analysis of the land use and soil type, geological maps were made of the study area using ArcGIS 10.0 software to lay out a grid of soil sampling points on a digital map of the Yanqi basin. All samples were acquired in July 2012 or July 2013 from a collection area. In order to best assess the ecological risk in the Yanqi basin, diverse land use types were encompassed in our study of salt ion distribution. Soil samples were collected at depths of 0–20 cm, where a hard plastic shovel was used to dig a vertical 20×20 cm soil profile. 1 kg uniform samples were collected, and then they were put into a clean cloth, numbered and sealed. The collection position, date, sample vegetation types and surrounding vegetation conditions of each sampling area were recorded. After the soil samples were taken back to the laboratory, they were air dried and impurities, such as plant residues and rocks, were removed. The samples were then pushed through a 20 mesh nylon sieve (0.84 mm) to eliminate the plant residue and stones. We then used agate to grind the soil samples through 100 mesh nylon sieves (0.25 mm) to prevent contamination and then stored the samples in plastic bottles [46].

Total soil salt content, soil salt ions, pH and EC tested are as follows: 50 g of ground sample were removed from the plastic bottles, dissolved in 250 ml of deionized water (CO_2_ has been removed) (1∶5, soil∶water) for 2 hours to fully dissolve salt ions contained in the soil. The samples were then put in a centrifuge tube, vibrated for 3 min with an oscillator and then centrifuged at a speed of 4,500–5,000 r·min^−1^. To get the supernatant prepared for analysis of total soil salt content, salt ions content, pH and EC, the method described by Lu (2000) was followed [44].

The total salt content of the soil was determined by gravimetry of the evaporation residue. First, the supernatant was absorbed in a porcelain dish, and hydrogen peroxide (H_2_O_2_) was used to ozidize organic matter. Then, the samples were boiled in a water bath at 105–110°C until it dried, and weighed. The drying quality of the residue is expressed as total salt content of the soil. The pH of the extracted supernatant was tested using a Potentiometric Titrimeter (G20, METTLER, and TOLEDO). Burette drive resolution was 1/20000. Mv/pH electrode measurement range was ±2000 mv. The ECs were tested using a Conductivity Meter (DDSJ-308A, Shanghai, China) with a measurement range of 0–1.999×10^5^ µs/cm and a test error of ±0.5% (FS) ±1.

The supernatant was run through a 0.45 µm drainage cellulose acetate membrane. Then, the cation content (K^+^, Na^+^, Ca^2+^, Mg^2+^) of the solutions was determined using an inductively coupled plasma atomic emission spectrometer (Vista MPX, Varian, USA). The anion content (Cl^-^, SO_4_^2-^, CO_3_^2-^, and HCO_3_^-^) of the solutions was determined using an Ion Chromatograph (ICS-90, Dionex, USA). All tests were conducted using the following protocol: a standard solution was prepared for Na^+^, K^+^, Ca^2+^, Mg^2+^, Cl^-^, SO_4_^2-^, CO_3_^2-^ and HCO_3_^-^. The salt ion content was determined by comparing each sample to the standard solution of known concentration. The standard solutions used for the salt ions in this study were national level standard material (Gss series, China). The coefficient of the best fitting curve was determined by the testing equipment based on the standard material and then the amount of the salt ions (Na^+^, K^+^, Ca^2+^, Mg^2+^, Cl^-^, SO_4_^2-^, CO_3_^2-^, and HCO_3_^-^) in the liquid supernatant was tested. After all the samples had been tested for their salt ion content (Na^+^, K^+^, Ca^2+^, Mg^2+^, Cl^-^, SO_4_^2-^, CO_3_^2-^, and HCO_3_^-^), we chose approximately 20% for retesting and found that 97.3% of the results were repeatable, inspiring confidence in the original data. After all the total salt ion contents (Na^+^, K^+^, Ca^2+^, Mg^2+^, Cl^-^, SO_4_^2-^, CO_3_^2-^, and HCO_3_^-^) of the solution were determined, we recalculated them from unit of µg/ml into mg/g (g/kg) using the method described by Bao (2005) [45]. To prevent contamination during the testing process, all glassware was soaked in 5% HNO_3_ for 24 hours, rinsed and then dried.

### Statistical analyses

#### Descriptive and multivariate statistical analysis

Descriptive statistical methods were used to analyze the range, mean, median, standard deviation, coefficient of variation, kurtosis and skewness of the total salt content, each salt ion, SAR, pH and EC of the soil samples. Correlation analysis, PC analysis and cluster analysis of the classic multivariate statistical method were used to process data and identify the soil salinity. Single factor analysis of variance (ANOVA) was used to analyze the differences in the amount of salt ions between different land use types. These analyses were all processed using the software SPSS 19.0.

#### Ordinary kriging method

Ordinary kriging (OK) is a commonly used linear spatial interpolation method that estimates variables at unsampled locations by using information from neighboring points and assigning weights to these points based on their distance from the point and the spatial variability structure. The OK method can be formulated as
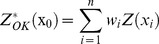
(1)where 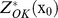 is the OK estimation at an unsampled location 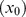, n is the number of samples in a search neighborhood, and 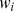 are the weights assigned to the *i*th observation 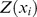. Weights are assigned to each sample such that the estimation or kriging variance 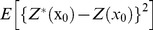 is minimized and the estimates are unbiased [47]. Weights are determined after computing a semivariogram that models spatial correlation and covariance structure between data points for each variable using Eq. 1 [48].
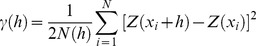
(2)where 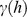 is the semivariance between two observation points 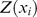 and 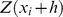 separated by a distance *h*, and N is number of observation pairs at the distance *h*.

Soil salinization evaluation criteria used in this research as under below (Table 1).

**Table 1:** Indicators used for risk assessment of soil salinization.

Indicators	Class limits and their ratings score				
	None	Slight	Moderate	Severe	Very severe
*EC (dS.m^−1^) [49]	<4	4–8	8–16	16–32	>32
**SAR [50]	<8	8–13	13–30	30–70	>70
Total salt content (%) (0–20 cm) [51]	<1	2∼3	3–4	4–8	>8

*EC is electrical conductivity; **SAR is sodium adsorption ratio.

## Results and Discussion

### Descriptive statistical analysis of soil salinity

The descriptive statistics concerning the soil properties in Yanqi basin in Table 2 show that the maximum and average values of HCO_3_^-^, Ca^2+^, CO_3_^-^, Na^+^, Mg^2+^, K^+^, SO_4_^2-^, Cl^-^, SAR, total amount of salts, EC, and pH were 0.98(0.19) g.kg^−1^, 1.95(0.75) g.kg^−1^, 0.85(0.46) g.kg^−1^, 2.43(1.19) g.kg^−1^, 1.89(0.58) g.kg^−1^, 2.13(0.69) g.kg^−1^, 1.58(1.14) g.kg^−1^, 2.36(0.98) g.kg^−1^, 33.241(10.51), 14.77(9.73) g.kg^−1^, 1.39(0.95) dS.m^−1^, and 8.55(8.15), respectively. Within the analysis of the soil samples, a large amount of variation occurred, suggesting that the sources and influencing factors of the salt properties in Yanqi basin are complex. This work found that the main salt ions accounted for 84.41% of the total salt content and were K^+^, Ca^2+^, Na^+^, Cl^-^, Mg^2+^ and SO_4_^2^. Meanwhile the amount of HCO_3_^-^ and CO_3_^-^ was very low. The pH of the soil of the study areas ranged from 7.85 to 8.55, which had only a small variation. However, there was a dramatic change within the total salt content of the soil, ranging from 1.16 to 14.77 g.kg^−1^.

**Table 2:** Descriptive statistics of the soil salt properties from Yanqi basin.

Elements	Ranges (g.kg^−1^)	Contributions(%)	Median (g.kg^−1^);EC (dS.m^−1^)	Average (g.kg^−1^)	Standard deviation (%)	Coefficient of variation (%)	Kurtosis (%)	Skewness (%)
HCO_3_^-^	0.13–0.98	3.51	0.171	0.19	12.25	35.37	0.14	0.58
CO_3_^-^	0.18–0.85	4.08	0.252	0.46	10.35	32.08	1.63	8.76
Ca^2+^	0.59–1.95	6.54	0.681	0.75	9.83	191.67	31.38	42.43
Na^+^	0.69–2.43	13.18	0.955	1.19	12.38	23.01	2.45	16.22
Mg^2+^	0.47–1.89	7.75	0.987	0.58	21.02	20.07	1.10	10.88
K^+^	0.49–2.13	12.36	0.855	0.69	23.04	226.25	23.47	51.81
SO_4_^2-^	0.93–1.58	18.95	1.245	1.14	12.56	19.63	1.46	0.76
Cl^-^	0.75–2.36	25.63	1.167	0.98	25.7	12.94	2.30	14.79
SAR	3.41–33.241	-	22.417	10.51	121.45	148.56	11.54	15.78
EC	0.7–1.39	-	0.981	0.95	15.09	21.27	1.46	10.99
pH	7.85–8.55	-	8.141	8.15	16.39	30.32	1.17	0.73
Total salt	1.16–14.77	-	8.56	9.73	15.36	126.73	16.84	18.46

The coefficient of variation is the ratio between the standard deviation and average, and it can be used to compare different dimensions of indicators. The coefficients of variation of HCO_3_^-^, CO_3_^-^, Na^+^, Mg^2+^, SO_4_^2-^, Cl^-^, EC, and pH were 35.37%, 32.08%, 23.01%, 20.07%, 19.63%, 12.94%, 21.27%, and 30.32%, respectively, and were of medium variation (10%<CV<100%). However, the coefficients of variation of SAR, the total amount of salts, Ca^2+^ and K^+^ were 148.56%, 126.73%, 191.67% and 226.25%, and, therefore, had high levels of variation (CV>100%)[52]. In particular, Ca^2+^ and K^+^ had higher coefficients of variation as compared to the other elements. From the perspective of skewness, the values of these ten soil properties are ordered as K^+^>Ca^2+^>total amount of salts>Na^+^>SAR>Cl^-^>EC>Mg^2+^>CO_3_^-^>SO_4_^2-^>pH>HCO_3_^-^.

### The differences in soil salt properties from different land use types and soil parent materials

**Figure 1:**
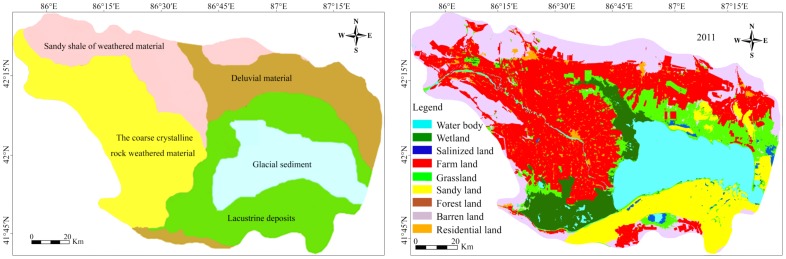
Land use types and parental material pattern in Yanqi basin.

Land utilization types and soil parent materials are the main examples of human activity and geological background that influence the salt ion content of soil. We analyzed the relationship between soil salt properties, human activity and geological background to further explore the distribution characteristics and sources of the soil salt properties of the Yanqi basin. The salt properties of the soil from each land use type and soil parent material of Yanqi basin are in Table 3. The land use types and soil parent materials of Yanqi basin are shown in Fig. 1. The analysis of these data suggests that the manner in which land is used has a significant influence on the amount of Ca^2+^ and K^+^. In farmland, the average content of Ca^2+^ was 1.83 g.kg^−1^, K^+^ was 1.71 g.kg^−1^, and the total amount of salts was 10.88 g.kg^−1^. This was significantly higher than in the other land use types including areas of urban construction, forest, grassland, urban construction areas and desert. Meanwhile the maximum average values of Na^+^, Mg^2+^, SO_4_^2-^, Ca^2+^, the total amount of salts, and K^+^ found in farmland were higher than in grassland, desert, and areas of urban construction. The variance test attained a significant level of 0.05, indicating these elements and their distribution are mainly controlled by human activity. The activities that had a significant influence on these soil salt properties include agricultural activities, such as irrigating, fertilizing and farming. The CV calculated indicates that the pH, EC, total amount of salts and all the soil salt ions measured belong to medium variability categories (10–100%). Among the five land use types, the differences between these groups were small and, therefore, the classes of element enrichment were not obvious.

**Table 3:** Statistical parameters of the soil salt ions found at 0–20 cm depth within the investigated land use and land cover categories of the study area.

LUCC SPM	Parameters	HCO_3_^-^ (g.kg^−1^)	CO_3_^-^ (g.kg^−1^)	Na^+^ (g.kg^−1^)	Mg^2+^ (g.kg^−1^)	K^+^ (g.kg^−1^)	Ca^2+^ (g.kg^−1^)	SO_4_^2-^ (g.kg^−1^)	Cl^-^ (g.kg^−1^)	Total salt (g.kg^−1^)	EC (dS.m^−1^)	pH
Farmland (n = 51)	Ranges	0.18–0.98	0.27–0.85	0.69–2.43	1.05–1.89	1.31–2.13	1.17–1.95	1.05–1.51	1.47–2.36	8.56–14.77	0.96–1.39	8.05–8.55
	Average	0.32a	0.62a	1.36a	1.15a	1.83a	1.71a	1.21a	1.68a	11.2a	1.02a	8.15a
	SD	22.45	15.23	31.35	23.3	27.57	37.73	24.57	19.19	23.97	18.64	14.77
Forest (n = 46)	Ranges	0.13–0.53	0.18–0.78	0.69–0.98	0.47–0.98	0.49–0.97	0.59–1.02	1.09–1.58	0.75–0.97	9.13–13.35	0.7–1.13	7.85-8.45
	Average	0.33a	0.41b	0.75b	0.87b	0.75a	0.77a	1.18b	0.82a	9.98b	0.94b	8.17b
	SD	14.75	17.73	23.53	25.55	23.74	23.73	17.86	28.33	16.79	18.71	14.37
Grassland (n = 63)	Ranges	0.21–0.73	0.25–0.82	1.03–1.63	0.54–1.02	0.61–1.31	0.89–1.14	0.93–1.19	0.96–1.61	9.08–11.24	0.76–1.25	7.92–8.42
	Average	0.42a	0.65b	1.32b	0.68b	1.18b	0.98b	1.04b	1.28b	9.45a	0.95b	8.27b
	SD	17.54	23.77	27.52	23.13	11.51	15.23	12.22	22.14	13.75	16.71	21.37
Desert (n = 71)	Ranges	0.26–0.83	0.19–0.76	0.98–1.57	0.47–1.44	0.95–1.56	0.99–1.19	1.05–1.24	1.08–2.29	9.31–13.89	0.78–1.34	8.06–8.44
	Average	0.58b	0.32b	1.31b	1.13b	1.21b	1.02b	1.11.6b	1.57b	10.56a	1.08b	8.28a
	SD	17.33	15.65	17.52	25.55	18.85	13.43	17.33	19.25	23.75	12.52	15.31
Urban construction areas (n = 40)	Ranges	0.49–0.93	0.33–0.83	0.91–1.54	0.65–1.03	0.51–1.51	0.88–1.21	0.94–1.18	0.79–1.31	6.16–14.13	0.75–1.33	7.94–8.51
	Average	0.53c	0.39c	1.21c	0.91c	0.97c	0.97c	1.06c	0.94c	10.52a	1.11c	8.31c
	SD	15.75	16.51	13.25	15.52	12.35	19.52	23.37	21.54	32.24	22.31	24.87
Sandy shale of weathered material (n = 66)	Ranges	0.13–0.86	0.18–0.38	1.19–2.39	0.47–1.75	0.58–2.11	0.59–1.36	0.93–1.53	0.75–2.36	8.69–14.77	0.79–1.26	7.86–8.55
	Average	0.45a	0.42a	1.51a	0.95a	0.91a	0.82a	1.01a	1.21a	10.88a	0.3a	7.93a
	SD	25.55	17.53	13.52	22.31	22.35	23.35	15.75	18.65	11.54	12.25	21.35
Coarse crystalline rock weathered material (n = 74)	Ranges	0.18–0.52	0.22–0.79	0.69–2.43	0.51–1.54	0.49–2.13	0.69–1.45	0.97–1.56	0.78–2.12	6.16–12.41	0.7–1.39	7.85–8.32
	Average	0.32b	0.38b	1.72b	0.81b	0.87b	0.95b	1.13b	1.46b	8.72a	1.14b	8.14b
	SD	13.35	11.41	21.25	23.74	11.29	12.52	21.57	22.53	15.54	12.57	24.58
Diluvial material (n = 68)	Ranges	0.21–0.93	0.24–0.85	0.81–2.23	0.65–1.89	0.64–1.72	0.71–1.56	0.95–1.58	0.85–2.26	7.89–13.25	0.84–1.28	7.89–8.19
	Average	0.67b	0.51b	1.42b	0.75b	0.98b	1.01b	1.05b	1.62b	9.82a	0.91b	8.01b
	SD	12.15	12.25	23.73	27.35	11.37	12.75	12.73	22.36	21.57	23.37	11.52
Lacustrine deposits (n = 63)	Ranges	0.33–0.98	0.23–0.79	0.85–2.35	0.52–1.49	0.71–1.98	0.62–1.95	0.96–1.51	0.96–2.19	8.98–11.51	0.85–1.32	7.97–8.37
	Average	0.74a	0.44b	1.16a	0.91c	1.02c	0.85c	1.06a	1.31b	9.24a	1.24a	8.23a
	SD	13.51	22.35	21.26	23.59	15.34	15.62	21.94	19.31	26.49	23.77	12.35
R^2^	LUCC (%)	31.21	9.74	13.49	8.4	71.45	37.85	11.3	10.8	56.71	12.64	12.43
	SPM (%)	58.79	34.59	16.47	28.93	32.7	11.4	17.9	15.9	23.51	17.53	15.2

Different small letters represent a significance of 0.05; LUCC represent land use types; SPM represent soil parent material types.

This research also found differences in the amount of organic soil salt ions. For example, HCO_3_^-^ reached a maximum in lacustrine deposits of 0.74 mg.kg^−1^. The maximum average values of Na^+^, Mg^2+^, K^+^, SO_4_^2-^, total amount of salts, and Cl^-^ were found in coarse crystalline rock weathered material, sandy shale of weathered material and lacustrine deposits, while the maximum average values of CO_3_^-^, Ca^2+^, and EC were found to be significantly higher in diluvial material than in sandy shale or coarse crystalline rock from weathered material or lacustrine deposits. For the five land use types, the class of element enrichment was not obvious as the differences between these groups were small.

R^2^ represents the ratio of the sum of squares in groups and the total error of the sum of squares. It reflects the contribution of different factors on the soil salt properties [53]. For this study, the land use types explained the variances in Ca^2+^ (37.85%), K^+^ (71.45%) and total amount of salts (56.71%), which were higher than that of the soil parent material. This demonstrates that the way land was used played a major role in the accumulation of Ca^2+^, K^+^ and total amount of salts in Yanqi basin. This analysis also found that the variances of HCO_3_^-^, CO_3_^-^, Mg^2+^, Na^+^, SO_4_^2-^, and Cl^-^ in the soil parent material are higher than those of the land use factors (Table 4), indicating that the soil parent material played a major role in the accumulation of these elements. However, there was little difference in the variances of EC and pH, indicating that these soil salt properties were mainly influenced by land use and soil parent material. Overall, the R^2^ analysis fits well with the results of the multivariate statistical analysis.

**Table 4:** The correction matrix of soil salt properties in Yanqi basin.

	EC	TSA	TSC	TS	Mg^2+^	Na^+^	K^+^	SO_4_^2-^	Cl^-^	CO_3_^-^	Ca^2+^	HCO_3_^-^	pH
EC	1												
TSA	0.58	1											
TSC	0.40	0.30	1										
TS	0.41	0.52**	0.38**	1									
Mg^2+^	0.10	0.04	0.11	0.06**	1								
Na^+^	0.98**	0.34	0.96**	0.95**	0.01	1							
K^+^	0.65	0.48	0.78*	0.71**	0.51	0.64	1						
SO_4_^2-^	0.87**	0.94**	0.42	0.93**	0.001	0.82**	−0.24	1					
Cl^-^	0.66**	0.55*	0.12	0.57**	0.13	0.69**	−0.15	0.24	1				
CO_3_^-^	0.48*	0.49	0.27	0.48**	−0.24	0.57	0.12	0.27	0.17	1			
Ca^2+^	0.42	0.55	0.51*	0.54**	0.22	0.25	0.57*	0.23	0.03	−0.14	1		
HCO_3_^-^	−0.25	−0.22	−0.20	0.21**	0.03	−0.16	−0.19	−0.42	0.28	0.16	−0.21	1	
pH	0.72**	0.62	0.14	0.82	0.42	0.32*	0.27	0.67**	0.58**	0.10	0.41	0.01	1

EC is electrical conductivity, TSA is the total anionic salt, TSC is the total cationic salt and TS is total salt content.

### Multivariable statistics

#### Correlation analysis

Table 4 shows the Pearson correlation coefficients between the soil salinity variables. There is a significant correlation of 0.96 (P<0.01) between the total amount of salt cations and Na^+^, as well as the total amount of salt cations and Ca^2+^ at 0.51 (P<0.05) in the soil in the Yanqi basin, but there is no significant correlation with other salt cations. Furthermore, we found a significant correlation between the amount of salt anions and SO_4_^2-^ of 0.94 (P<0.01), and the correlation coefficients between the total amount of salt anions and Cl^-^ or CO_3_^2-^ are 0.55 and 0.49 (P<0.05), respectively. This indicates that SO_4_^2-^ was the primary salt anion, Cl^-^ was the secondary and CO_3_^2-^ was the tertiary. Together, the correlation coefficients between the total amount of salts and Cl^-^, and the total amount of salts and SO_4_^2-^ are 0.57 and 0.92, respectively (P<0.01), indicating that the main types of salt in the soil were sulfate and chloride. This study also found that there are close correlation coefficients between the EC and Na^+^, the EC and Cl^-^, and the EC and SO_4_^2-^ content, where the coefficients are 0.98, 0.66, and 0.87, respectively (P<0.01). Additionally, the correlation coefficient between the soil EC and CO_3_^2-^ is 0.48, which is also significant (P<0.05). Overall, a significant correlation was found between pH and EC, and total amount of salts (TS) and salt anions (Na^+^, Ca^2+^, Ca^2+^, Ca^2+^, Cl^-^, SO_4_^2-^, HCO_3_^-^, and CO_3_^2-^) (P<0.01). Further analysis shows that the correlation coefficient between the pH and SO_4_^2-^ is 0.67 and between pH and Cl^-^ is 0.58 (P<0.01), indicating the pH of the soil is primarily influenced by the SO_4_^2-^ and Cl^-^ content.

#### Principal component analysis and clustering analysis

**Figure 2:**
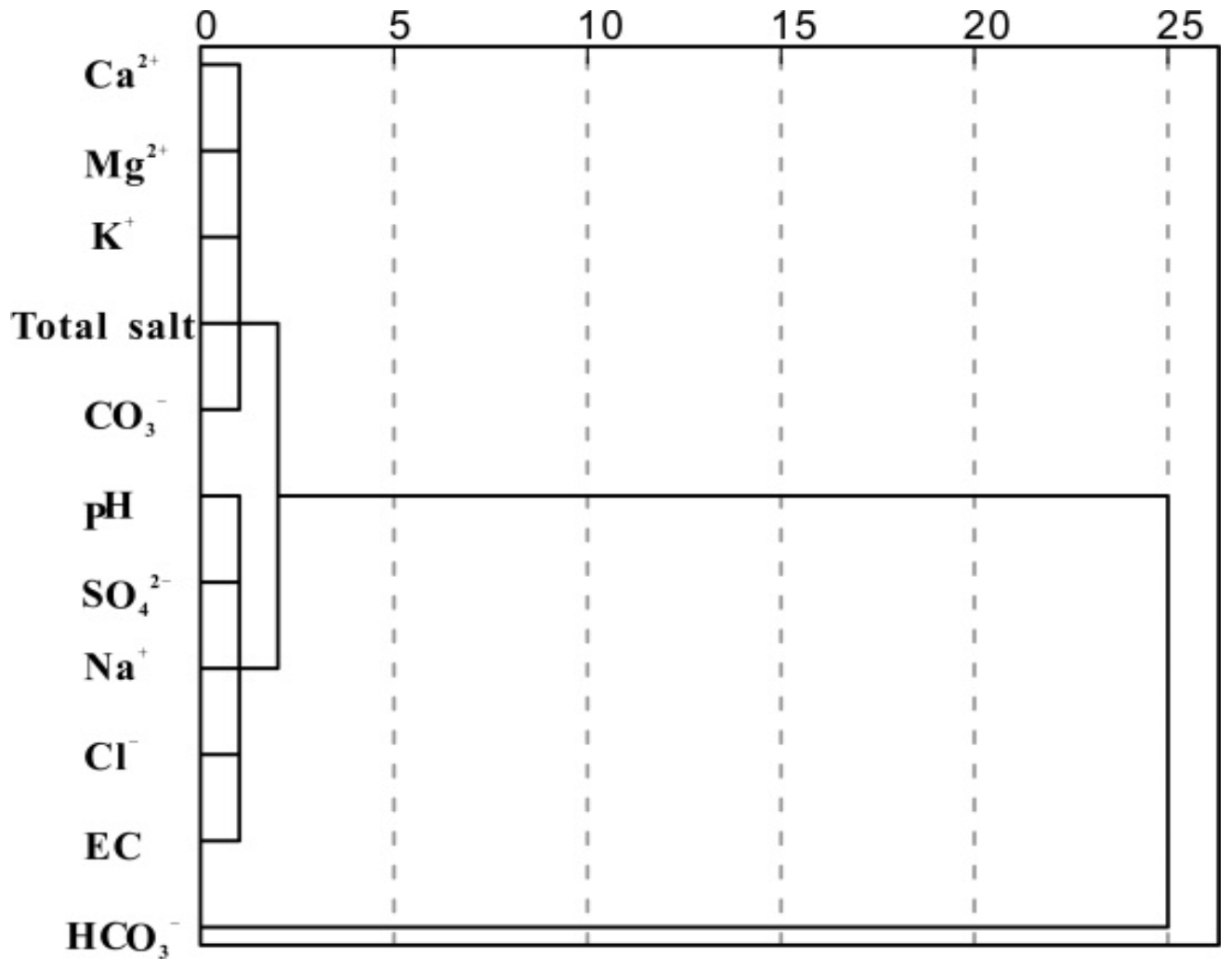
Clustering tree of soil salt properties of Yanqi basin.

All of the elements studied were found to fall into four PCs (Table 5) with a cumulative variance of 95.72%. This is a reflection of their sources and main influences, particularly for the ten indicators of Cl^-^, Na^+^, EC, pH, K^+^, Mg^2+^, Ca^2+^, HCO_3_^-^, CO_3_^2-^, total amount of salts, and SO_4_^2-^. Among these, the variance contribution rate of the first PC (Cl^-^, Na^+^, SO_4_^2-^, EC and pH) was 33.75% and the second PC (Ca^2+^, K^+^, Mg^2+^, and total amount of salts) was 28.54%. The primary contribution was by agricultural development, in particular from herbicide application and acid salt fertilizer [54], [55]. The variance contribution rates of the third (CO_3_^-^) and fourth PCs (HCO_3_^2-^) were at 18.25% and at 15.18%, respectively. Upon combining the sampling sites within their respective land use types, it was found that the samples that had a high content of salts were often taken from the desert, grassland and forest, and appear to have originated from natural sources. This analysis also showed there were larger loads of Ca^2+^ in the first PC, SO_4_^2-^ in the second PC and K^+^, total salt content in the fourth PC, indicating these elements were influenced by both artificial and natural sources. We used clustering analysis to see if the results are consistent with the results from the PC analysis (Fig. 2). All the elements were classified into four categories: the first category consisted of Cl^-^, Na^+^, SO_4_^2-^, pH, and EC; the second of Mg^2+^, Ca^2+^, K^+^, and the total amount of salts; the third of HCO_3_; and the fourth of CO_3_^2-^.

**Table 5:** Factors matrix of soil salt properties from Yanqi basin.

Soil salt properties	Principal components			
	PC 1	PC 2	PC 3	PC 4
K^+^	−0.18	0.65	0.37	0.56
Mg^2+^	0.37	0.80	−0.07	0.14
Ca^2+^	0.63	0.74	0.50	0.45
CO_3_^2-^	−0.05	−0.02	0.59	−0.10
SO_4_^2-^	0.61	0.54	0.42	0.17
pH	0.49	0.15	0.06	−0.79
Cl^-^	0.91	−0.04	0.29	0.13
Na^+^	0.68	−0.08	0.56	0.31
EC	0.46	−0.01	−0.73	0.13
HCO_3_^-^	−0.09	−0.09	0.29	0.37
Total salt	0.21	0.78	0.14	0.62
Percentage of variance (%)	33.75	28.54	18.25	15.18
Percentage of cumulative variance (%)	33.75	62.29	80.54	95.72

#### Spatial distribution of soil salt ions in Yanqi basin

**Figure 3:**
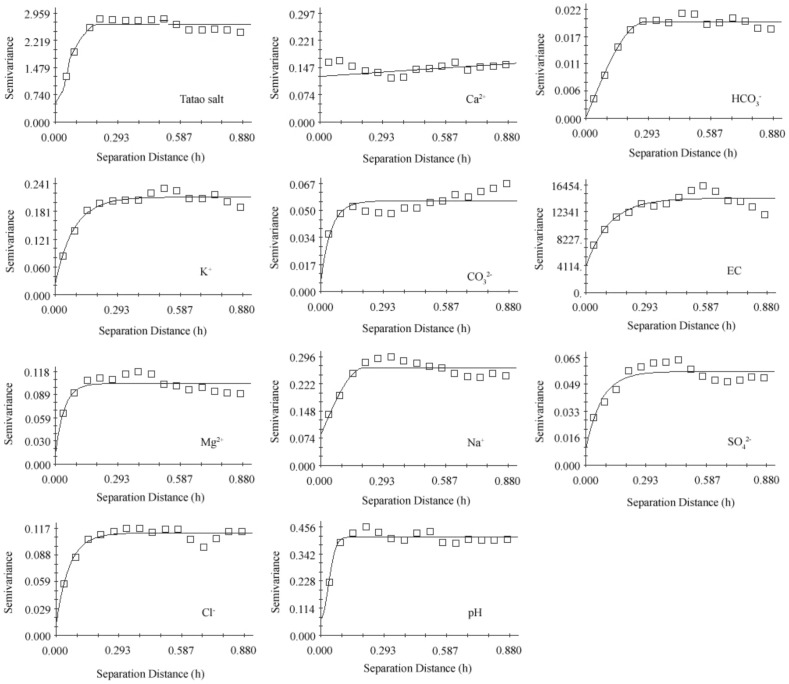
The semivariance function diagram of the soil salt properties of Yanqi basin.

The spatial dependence of the soil salt properties was determined by semivariance analysis in order to quantitate the spatial variability. The parameters of the semivariogram included model type, nugget, sill and effective range. The nugget value (C_0_) represents the random variation derived from measurement inaccuracy or variations in properties that cannot be detected in the sample range [54]. The sill value is the upper limit of the fitted semivariogram model [47]. The ratio of nugget to sill was a criterion to classify the spatial dependence of soil properties and it reflects the influence of regional factors (nature) and the role of the non-regional factors (human factors). The range of the semivariogram (A_0_) represents the average distance through which the variable semivariance reaches its peak value. A small effective range implies a distribution pattern composed of small patches. The cross-validation value is the coefficient of determination (R^2^) of the correlation between the measured values and the cross-validation values, which were predicted based on the semivariogram and neighbor values [53]. Previous studies have shown that the semivariogram often differs considerably from its regional counterpart [33], [53]. Fig. 3 and Table 6 show the tested variables over the study area that were modeled using spherical, gaussian and exponential semivariograms with the lower nugget effect based on the coefficients of determination (R^2^) and residual sum of square (RSS). In this respect, a low ratio (less than 25% as was found for pH, Ca^2+^, Mg^2+^, and HCO_3_^-^) means that a large part of the variance is introduced spatially. This implies a strong spatial dependency of the variable, most likely due to intrinsic factors, including soil formation factors, such as soil parent materials, topography, and/or climate. A high ratio (more than 75% was found with Na^+^ and Cl^-^) often indicates a weak spatial dependency in the present sampling that was most likely due to extrinsic factors, including contamination, irrigation, and soil management practices, where the fertilization and the soil chemical properties were continuously shifting. In this research the Nug/Sill ratios of CO_3_^2-^, and total salt content are 67.27 and 67.53% in the range between 25% and 75%, indicating the total salt content of the soil in Yanqi basin was influenced by the above factors. Additionally, all the other variables have a moderate spatial dependency for both intrinsic and extrinsic factors. The effective range calculated for variograms of different soil salt properties was 50–280 m, indicating that the sample distance was adequate for the characterization of the spatial variability of these properties.

**Table 6:** The spatial variation parameters of the soil salt properties of Yanqi basin.

Soil salt properties	Model	Nugget (Co)	Sill (Co+C)	Nug/Sill ratios C_0_/(C_0_+C) (%)	Range A_0_ (m)	R^2^	RSS
Na^+^	Spherical	0.084	0.102	82.35	200	0.921	4.053E-03
K^+^	Exponential	0.068	0.212	32.08	90	0.861	2.900E-03
Ca^2+^	Exponential	0.034	0.217	15.67	101	0.954	1.131E-02
Mg^2+^	Spherical	0.023	0.103	22.33	100	0.733	1.288E-03
Cl^-^	Gaussian	0.087	0.111	78.38	100	0.891	4.104E-04
CO_3_^2-^	Exponential	0.037	0.055	67.27	50	0.914	4.014E-04
HCO_3_^-^	Spherical	0.004	0.021	19.05	280	0.927	2.446E-05
SO_4_^2-^	Exponential	0.012	0.041	29.27	80	0.823	1.826E-04
pH	Gaussian	0.071	0.415	17.11	50	0.872	5.215E-03
EC	Exponential	0.082	0.106	77.36	120	0.791	2.081E-05
Total salt	Spherical	0.497	0.836	59.45	180	0.833	1.472E-02

The main application of geostatistics in soil science has been estimating and mapping the chemical properties in the soil of unsampled areas. Maps for each of the soil properties can be obtained using an ordinary kriging interpolation based on the best-fit semivariogram model. The skewness, defined as more than +1 or less than -1, indicates that some soil properties were not normally distributed. For these properties, it is difficult to estimate the semivariogram and doing so would result in a high value of kriging standard deviations [53]. Lognormal kriging with non-linear transformations is an alternative method for dealing with a data set with outliers or a non-normal distribution [31], [33]. Since the concentration of the variables with the non-normal distribution had a lognormal distribution, their concentrations were log-transformed, resulting in more regular variograms. The kriging interpolations were performed on the log-concentrations and the estimated values were back-transformed by an exponential function. As seen in the results, total amounts of salt, pH, EC, and the concentrations of Na^+^, K^+^, Mg^2+^, CO_3_^2-^, and Cl^-^ were relatively higher when from farmland and grassland. The spatial patterns of these variables had a significant geographical distribution with their primary occurrence in the western, north and central areas, which were higher than in other areas. This further proves the effect of the intrinsic factors of topography, soil forming factors and soil type, and extrinsic factors of soil management practices, such as fertilization, use of organic fertilizer and land management in farms, on the spatial distribution of soil chemical properties [33], [53]. The soil salinity tended to increase from the margin to the center across the study area, where agricultural wells are denser. It is clear that substantial soil salinization has taken place in these areas due to the effects of land management, farming and climate conditions, such as rainfall and high evaporation. This shows that more attention should be paid to these areas to prevent future problems. Soil pH in the study area ranged from 7.95 to 8.55 in most parts of the Yanqi basin. This pH range falls into the middle level of values that meets the fundamental conditions for plant growth and fertility. The ECs of the soil in all sections were within the acceptable value of <4 dS/m as based on the soil quality standard given by Bao et al. [45]. Areas presently not affected by salinity, but near saline areas, are potential areas for the development of salinity in the future, especially if they are also low-lying. In this respect, it is important to take necessary precautions and implement proper land use plans and cultivation practices.

#### Assessment of soil salinization risk in Yanqi basin

**Figure 4:**
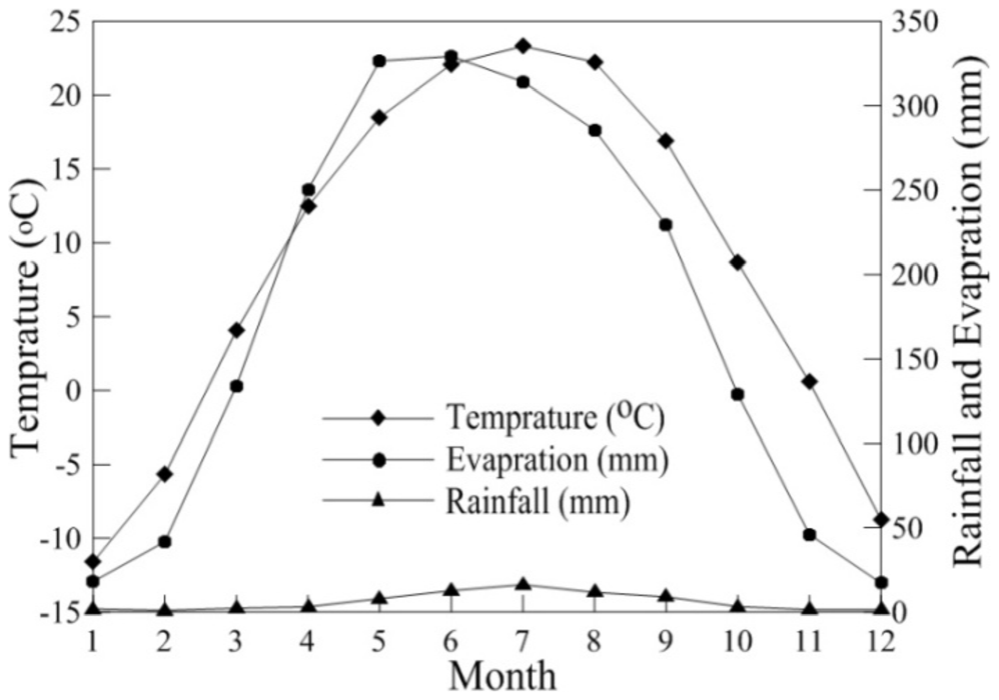
The monthly evaporation, precipitation and temperature of Yanqi basin in 2011.

Soil salinization occurs mainly in arid and semi-arid regions. It may arise due to climate, but is more likely to occur when irrigation practices alter the natural salt balance. Irrigation promotes soil salinization by raising the water table of the underlying aquifer, thus carrying salts upwards. Salts, unlike water, remain in the soil as evaporation and plant transpiration take place, thereby amplifying the salinity. The accumulation of salts in the surface and near-surface zones of soil is a major issue of environmental degeneration and is one of the main causes of low crop yields, loss of land and decreased production. The risk of soil salinization in the Yanqi basin is presented in Table 7. We observed that the whole area has a low salinization risk, as previously mentioned, mainly due to anthropogenic activities and climatic variation [39], [40]. The overall salinization risks for farmland, grassland, forest, urban construction areas and desert were none, none, none, low, and moderate, respectively. The mean values of the salinization risk indicators, except the SAR in farmland and desert, are lower than the corresponding maximum limitations that are predicted for the risk (Table 7). This indicates that farmland and desert in the study area have a low salinization risk, but they have mean values of 15.2 and 32.7 for SAR, which showed a moderate grade of salinization (Table 1, Table 7). This indicates a potential risk for environment declination. This research showed the grassland and forest have no risk of salinization, while the areas of urban construction have a moderate risk of salinization. In terms of spatial distribution, the soil salinization risk and the soil salt ions present at higher concentrations in the study area were similar. Environment declination due to soil salinity within the study area almost all took place on the edge of a lake, pond or river. These areas are heavily affected by climate warming, which in turn results in an increase in the amount of evapotranspiration that exceeds 2438.9 mm/a and in the average annual rainfall <100 mm (the drought and waterlog) (Fig. 4). Apart from natural factors, the main driving factors that jointly determined how local dwellers changed the landscape pattern were land use policies, economic systems and population growth. Human activity increases salinization through excessive application of irrigation water without adequate drainage. In addition, the cultivation of grassland is another major cause of inland salinization [40], [41]. On one side of the study area, plenty of grass landscape suffered damage, and, in some regions, the land salinization and desertification problem were serious enough to destroy the harmony between the material cycle and energy flow of the ecosystems [42]. On the other side of the study area, the habitat for wildlife was deteriorating, thus seriously threatening the biological diversity. Therefore, establishing and modifying policy, adjusting the irrigation system, improving drainage, dredging the surface water system, promoting circulation between surface water and underground water, and setting up wetland resource monitoring systems are necessary in order to restore damaged wetland and grassland [49], [50], [55].

**Table 7:** Classification of soil salinization risk taken at 0–20 cm depth within the investigated land use and land cover categories in Yanqi basin.

LUCC categories	EC	Total salt content	SAR	Levels of risk
Farmland	1.981	0.552	15.2	Low
Grassland	0.901	0.715	7.5	None
Forest	0.857	0.382	6.8	None
Urban construction areas	1.035	2.761	8.2	Low
Desert	1.024	3.657	32.7	Moderate

## Conclusions

(1) From all the soil samples taken in this study, it can be gathered that there are numerous salt properties that vary largely between different samples. This analysis shows that the main salt ions in the soil were K^+^, Ca^2+^, Na^+^, Cl^-^, Mg^2+^, and SO_4_^2-^, which accounted for 84.41% of the total salt content of the soil samples. Conversely, the concentrations of HCO_3_^-^ and CO_3_^-^ were very low. Except for the high variation found for the amounts of Ca^2+^ and K^+^ (191.67% and 226.25%, respectively), the other soil salt properties of Yanqi basin had moderate levels of variation (10%<CV<100%).

(2) From the analysis shown that the average values of Na^+^, Mg^2+^, SO_4_^2-^, Ca^2+^, total amounts of salt and K^+^ being higher in farmland than grassland, desert, and urban construction areas. This work also determined that the maximum average values of Na^+^, Mg^2+^, K^+^, SO_4_^2-^, total amount of salts, and Cl^-^ were found in coarse crystalline rock weathered material, sandy shale of weathered material and lacustrine deposits. Within the five land use types examined, they were not obvious which classes of elements were enriched and the differences between these groups were small.

(3) PC analysis determined that PC1 (Cl^-^, Na^+^, SO_4_^2-^, EC, and pH) and PC2 (Ca^2+^, K^+^, Mg^2+^, and total amount of salts) originated from artificial sources, while PC3 and PC4 (CO_3_^-^ and HCO_3_^2-^) originated from natural sources. Together, this research shows that Ca^2+^, K^+^, SO_4_^2^ and the total amount of salts were influenced by both artificial and natural sources. Clustering analysis is consistent with the results from the PC analysis.

(4) From the geo-statistical point of view, it can be speculated that pH and soil salt ions, such as Ca^2+^, Mg^2+^ and HCO_3_^-^, had a strong spatial dependency. Meanwhile, Na^+^ and Cl^-^ had only a weak spatial dependency, which was probably due to extrinsic factors, such as contamination, irrigation, and current soil management practices. We evaluated the EC, SAR and total salt content standard to reveal the risk of soil surface salinization. Soil salinization indicators suggest that the entire area had a low risk of salinization as mentioned previously, and this risk was mainly due to anthropogenic activities and climate variation. It is recommended that management of salinized land be preceded by an assessment of local factors and processes that may affect land composition.

Although the overall soil environment was healthy in the Yanqi basin, human activity, such as excessive groundwater pumping, have negatively impacted conditions by inducing soil salinization in the oasis. This matter deserves increased attention. This study can be considered an early warning of soil salinization and alkalization in the Yanqi basin. It can also provide a reference for environmental protection policies and for rational utilization of land resources in the arid region of Xinjiang, northwest China, as well as for other oases of arid regions in the world.
